# Investigation of Changes in Irisin and Nesfatin-1 Levels in Chronic Viral Hepatitis

**DOI:** 10.3390/jcm15093209

**Published:** 2026-04-23

**Authors:** Feray Ferda Senol, Ilkay Bahcecı, Sermin Algül, Ozlem Aytac, Arzu Şenol, Yusuf Çelik

**Affiliations:** 1 Department of Medical Microbiology, Elazığ Fethi Sekin City Hospital, 23280 Elazığ, Turkey; drferdasenol@yahoo.com (F.F.S.); ozlemozlem5@hotmail.com (O.A.); 2 Department of Medical Microbiology, Faculty of Medicine, Recep Tayyip Erdoğan University, 53100 Rize, Turkey; 3 Department of Physiology, Van Yüzüncü Yıl University, 65090 Van, Turkey; serminalgul@yyu.edu.tr; 4 Infectious Diseases and Clinical Microbiology, Elazığ Fethi Sekin City Hospital, 23280 Elazığ, Turkey; arzu.senol@saglik.gov.tr; 5 Biostatistics Department, Medical Faculty, Biruni University, 34015 Istanbul, Turkey; ycelik@biruni.edu.tr

**Keywords:** chronic hepatitis B, chronic hepatitis C, HBeAg-negative chronic infection, irisin, nesfatin-1, biomarker

## Abstract

**Background**: Chronic viral hepatitis is a major global health problem associated with progressive liver injury and an increased risk of cirrhosis and hepatocellular carcinoma. The identification of novel biomarkers may improve disease monitoring and diagnostic accuracy. **Methods**: In this prospective case–control study, a total of 90 participants were included: 20 patients with chronic hepatitis B (CHB); 20 with chronic hepatitis C (CHC); 20 with HBeAg-negative chronic infection (HCI); and 30 age-, sex-, and body mass index-matched healthy controls. Serum irisin and nesfatin-1 levels were measured using enzyme-linked immunosorbent assays (ELISAs). Group comparisons were performed using multivariate analysis of variance (MANOVA) followed by Scheffé post hoc tests. Receiver operating characteristic (ROC) curve analysis was used to evaluate diagnostic performance. **Results**: Significant differences were observed among groups in terms of irisin, nesfatin-1, total bilirubin, and platelet counts (*p* ≤ 0.05). Nesfatin-1 levels were significantly higher in all patient groups compared with healthy controls (*p* < 0.001). Irisin levels were only significantly lower in the HCI group (*p* < 0.001). ROC analysis indicated that nesfatin-1 may have the potential to discriminate between infected patients and healthy individuals; however, the generalizability of this finding is limited by the study design and sample size. **Conclusions**: Nesfatin-1 may represent a potential biomarker for chronic viral hepatitis, whereas alterations in irisin levels may be more specific to the inactive carrier phase.

## 1. Introduction

The liver plays a crucial role in numerous physiological processes, including digestion, metabolism, detoxification, immune regulation, and the maintenance of energy homeostasis. It is also a key organ in lipid and glucose metabolism. However, viral infections and chronic inflammatory processes may impair hepatic function and ultimately lead to severe complications, such as cirrhosis and hepatocellular carcinoma (HCC).

Infections caused by hepatitis B virus (HBV) and hepatitis C virus (HCV) represent two of the most common causes of chronic liver disease worldwide. It is estimated that approximately 328 million individuals are chronically infected with HBV or HCV. A comprehensive meta-analysis including 127 studies reported global seroprevalence rates of 5.8% for hepatitis B surface antigen (HBsAg) and 10.3% for anti-HCV antibodies [1]. In addition to viral factors, metabolic conditions—particularly insulin resistance—have been shown to contribute to disease progression and to increase the risk of hepatocellular carcinoma among individuals with chronic viral hepatitis [2,3].

HBV and HCV infections affect lipid metabolism through distinct mechanisms. HBV primarily exerts indirect effects via viral proteins; in particular, the HBV X protein has been reported to modulate the expression of host genes involved in lipid synthesis and transport, thereby contributing to metabolic alterations [2]. In contrast, HCV may directly contribute to hepatic steatosis. Genetic variations in microsomal triglyceride transfer protein have also been associated with hepatic steatosis in patients with HCV infection [4].

In individuals with pre-existing metabolic dysfunction-associated steatotic liver disease (MASLD), the subsequent acquisition of HBV or HCV infection may further accelerate disease progression. In this setting, metabolic disturbances—particularly insulin resistance and lipotoxicity—contribute to the formation of a pro-inflammatory hepatic environment, which enhances virus-related hepatocellular damage and promotes fibrogenesis [5,6]. In particular, insulin resistance has been associated with increased hepatic de novo lipogenesis, and may facilitate HCV replication while simultaneously reducing treatment efficacy, especially in interferon-based therapies [5,6].

Irisin, a peptide hormone generated by the proteolytic cleavage of fibronectin type III domain-containing protein 5 (FNDC5), has recently attracted attention as a potential biomarker in metabolic and inflammatory diseases. FNDC5 consists of a signal peptide, two fibronectin type III domains, and a C-terminal transmembrane domain. Following cleavage, irisin is released into circulation and exerts various physiological effects. Its synthesis is mainly stimulated by physical activity and is regulated by peroxisome proliferator-activated receptor gamma coactivator-1 alpha (PGC-1α), a transcriptional coactivator highly expressed in metabolically active tissues such as skeletal muscle, heart, liver, and brown adipose tissue [6,7,8]. Since the seminal study by Boström et al. in 2012, irisin has rapidly become a major focus of research and has been associated with various metabolic disorders and liver diseases [8,9]. Recent animal studies have suggested that exogenous administration of recombinant irisin or overexpression of the irisin-encoding gene (FNDC5) may ameliorate hepatic steatosis and steatohepatitis, partly by restoring impaired autophagy, enhancing fatty acid oxidation, and preventing cytokine-mediated hepatocyte apoptosis [10].

Nesfatin-1, an 82-amino-acid peptide derived from nucleobindin-2 (NUCB2), has been demonstrated to have a wide tissue distribution, particularly in the central nervous system as well as in peripheral tissues such as adipose tissue and the gastrointestinal tract [11]. Although its specific receptors have not yet been fully identified, previous studies suggest that G-protein signaling as well as protein kinase A and protein kinase C pathways may mediate its biological effects [12,13]. Nesfatin-1 has been implicated in the regulation of multiple metabolic processes, including appetite suppression, reduction in white adipose tissue mass, decreased gastric motility, and reductions in circulating triglycerides, cholesterol, lipid levels, and blood glucose concentrations [13,14].

Despite its widespread distribution throughout the body, the mechanisms underlying the biological actions of nesfatin-1 remain incompletely understood. Several attempts have been made to identify specific intracellular or extracellular receptors; however, these efforts have yielded inconclusive results. Nevertheless, accumulating evidence suggests that nesfatin-1 may interact with a specific receptor mediating its physiological functions. Autoradiographic studies using iodine-125-labeled nesfatin-1 have demonstrated binding sites in both central and peripheral tissues, including the brain, stomach, duodenum, jejunum, ileum, and pancreas, whereas no binding activity was detected in the colon [15]. Furthermore, a potential interaction with G protein-coupled receptors has been suggested, as nesfatin-1 has been shown to increase intracellular Ca2+ levels in hypothalamic neurons, likely involving L-, P-, and Q-type calcium channels [16].

Although irisin and nesfatin-1 have been investigated in various metabolic and inflammatory conditions, their roles in liver physiology and pathology remain insufficiently characterized. In particular, no previous study has systematically evaluated serum levels of these peptides in patients with chronic hepatitis B virus (HBV) or hepatitis C virus (HCV) infection in the absence of cirrhosis or hepatocellular carcinoma. Therefore, the aim of this study was to evaluate serum irisin and nesfatin-1 levels in individuals with chronic hepatitis B (CHB), HBeAg-negative chronic infection (HCI), and chronic hepatitis C (CHC) compared with healthy controls, and to assess their potential value as novel biomarkers for chronic viral hepatitis.

## 2. Materials and Methods

### 2.1. Study Population

A total of 90 participants were enrolled, including 60 patients and 30 healthy controls, who presented to the Infectious Diseases Outpatient Clinic of Elazığ Fethi Sekin City Hospital between 12 May 2024 and 31 December 2024. The patient group consisted of 20 individuals with chronic hepatitis B (CHB), 20 with chronic hepatitis C (CHC), and 20 with HBeAg-negative chronic infection (HCI).

### 2.2. Inclusion and Exclusion Criteria

Eligible participants were individuals diagnosed with CHB, CHC, or HCI who had not received prior antiviral therapy.

Exclusion criteria included alcohol consumption, diabetes mellitus, non-alcoholic fatty liver disease (NAFLD), thyroid disorders, renal disease, pregnancy or menopause, and malignancies. Individuals receiving steroids, metformin, or statins were also excluded.

In addition, both patient and control groups consisted of individuals with comparable and generally moderate levels of physical activity, based on clinical evaluation and self-reported information, in order to minimize potential metabolic variability between groups.

The control group consisted of healthy individuals without chronic disease, viral hepatitis, or the conditions listed above. Controls were matched with patients according to age, sex, and body mass index (BMI). None of the participants had hepatocellular carcinoma (HCC) or liver cirrhosis.

### 2.3. Diagnostic Criteria and Viral Load Quantification

Patients were classified into three diagnostic categories:

- Chronic Hepatitis B (CHB): Persistence of HBsAg for ≥6 months, anti-HBc IgG positivity, HBeAg positivity or negativity, and serum HBV DNA levels between 2000 and 20,000 IU/mL.
- Chronic Hepatitis C (CHC): Positive anti-HCV serology with detectable HCV RNA confirmed by reverse transcription polymerase chain reaction (RT-PCR).
- HBeAg-negative Chronic Infection (HCI): HBeAg-negative chronic infection (HCI) was defined as persistence of HBsAg for ≥6 months, HBeAg negativity, anti-HBc IgG positivity, and low-level viremia, in accordance with international clinical guidelines [17,18], typically characterized by HBV DNA levels <2000 IU/mL and normal ALT levels. In the present study, all patients classified as HCI had HBV DNA levels below the lower limit of quantification (<31.6 IU/mL) of the real-time PCR assay used, indicating very low or undetectable viral replication.

Viral loads were quantified using commercial assays:

- HBV DNA: COBAS AmpliPrep/COBAS TaqMan HBV Test (Roche Diagnostics, Basel, Switzerland), limit of detection (LOD) ≈ 20 IU/mL and limit of quantification (LOQ) = 31.6 IU/mL.
- HCV RNA: COBAS TaqMan HCV Test v2.0 (Roche Diagnostics, Basel, Switzerland), LOD = 15 IU/mL and LOQ = 25 IU/mL.

Diagnostic thresholds and classifications followed the recommendations of the European Association for the Study of the Liver (EASL) and the American Association for the Study of Liver Diseases (AASLD) guidelines [17,19].

### 2.4. Blood Sampling and Processing

Venous blood samples (5 mL) were collected after overnight fasting. Samples were centrifuged at 3000 rpm for 10 min, and serum was separated within 30 min of collection. All serum samples were stored at −80 °C and subjected to no more than one freeze–thaw cycle before analysis.

### 2.5. Biochemical and Hematological Analyses

Biochemical parameters were measured using a Beckman Coulter AU5800 automated analyzer (Beckman Coulter, Brea, CA, USA). Hematological analyses were performed using the Beckman Coulter DxH 800 hematology analyzer (Beckman Coulter, USA). Coagulation parameters, including prothrombin time (PT) and international normalized ratio (INR), were analyzed using the Sysmex CS coagulation analyzer (Sysmex Corporation, Kobe, Japan).

### 2.6. ELISA Measurement of Irisin and Nesfatin-1

Serum concentrations of irisin and nesfatin-1 were measured using commercially available ELISA kits (SunRed Bio, Shanghai, China):

- Irisin: Catalog No. 201-12-5328
- Nesfatin-1: Catalog No. 201-12-4341

All assays were performed using the Triturus automated ELISA processor (Grifols, Barcelona, Spain).

#### Assay Validation

Calibration curves were generated using seven standards ranging from 0.1 to 100 ng/mL, with a validated working range of 0.5–50 ng/mL. The limit of detection (LOD) for both markers was 0.3 ng/mL, while the limit of quantification (LOQ) was 0.5 ng/mL.

Precision analysis demonstrated an intra-assay coefficient of variation (CV) below 8% and an inter-assay CV below 12%. Acceptance criteria included a standard curve coefficient of determination (R^2^) ≥ 0.98 and duplicate sample CV ≤ 15%. Samples not meeting these criteria were reanalyzed. All measurements were performed in duplicate.

Laboratory operators were blinded to the group assignments. Considering the reported variability in commercial irisin assays, the selected kits were chosen based on previous validation studies and consistent internal quality controls to ensure reliability for comparative analyses.

Artificial intelligence (AI)-assisted tools were used solely for language editing and grammar improvement during manuscript preparation. No AI tools were used for data analysis, statistical evaluation, or interpretation of results. The authors take full responsibility for the content of the manuscript.

### 2.7. Ethical Approval

The study protocol was reviewed and approved by the Ethics Committee of Fırat University (Approval No. 2024/07-30, dated 9 May 2024). Written informed consent was obtained from all participants prior to enrollment. All procedures performed in this study were conducted in accordance with the ethical standards of the institutional research committee and with the principles of the Declaration of Helsinki.

### 2.8. Statistical Analysis

Continuous variables were expressed as a mean ± standard deviation (SD). The normality of data distribution was assessed using the Kolmogorov–Smirnov test.

Group comparisons were performed using multivariate analysis of variance (MANOVA), followed by Scheffé post hoc tests for pairwise comparisons. Receiver operating characteristic (ROC) curve analysis was conducted to evaluate the diagnostic performance of serum irisin and nesfatin-1 levels. The area under the ROC curve (AUC) was used as a measure of diagnostic accuracy and interpreted as follows: >0.9, excellent; 0.8–0.9, very good; 0.7–0.8, good; 0.6–0.7, fair; and <0.6, poor.

All statistical tests were two-sided, and a *p*-value ≤ 0.05 was considered statistically significant. Statistical analyses were performed using R statistical software (version 3.6.2; R Foundation for Statistical Computing, Vienna, Austria).

### 2.9. Power Analysis and Sample Size

Based on previous literature, nesfatin-1 has demonstrated a moderate discriminative performance (approximately 0.74) in distinguishing patients from healthy controls [18]. In the present study, this performance was hypothesized to improve to 0.87. Assuming a confidence level of 95% and a statistical power of 80%, the required sample size was calculated as 90 participants. Sample size estimation was performed using R statistical software (version 3.6.2; R Foundation for Statistical Computing, Vienna, Austria).

## 3. Results

The demographic and baseline clinical characteristics of the study population are presented in Table 1. No significant differences were observed among the groups with respect to age, sex distribution, or body mass index (BMI) (*p* > 0.05), indicating comparability between the patient and control cohorts.

According to the MANOVA test results, there was a significant difference in irisin and nesfatin-1 levels among the study groups (Wilks’ Lambda = 0.15; F = 5.630; *p* < 0.001). The MANOVA test results for the groups are presented in Table 2.

According to the results of Table 2, significant differences were found between the patient groups and the control group in terms of irisin, nesfatin-1, total bilirubin, and PLT tests (*p* < 0.05). Following significant MANOVA results, Scheffé post hoc tests were performed for pairwise comparisons. Due to the presence of significant differences, a Scheffé post hoc test was conducted as the second stage of the MANOVA test for multiple comparison analysis. The results of this analysis, showing only the significantly different groups, are presented in Table 3.

Based on the MANOVA analysis, statistically significant differences were observed among the groups in terms of irisin, nesfatin-1, total bilirubin, and PLT parameters (*p* < 0.05). Following these findings, Scheffé post hoc analysis was performed to identify pairwise differences between the groups, and the results are presented in Table 4.

**Figure 1:**
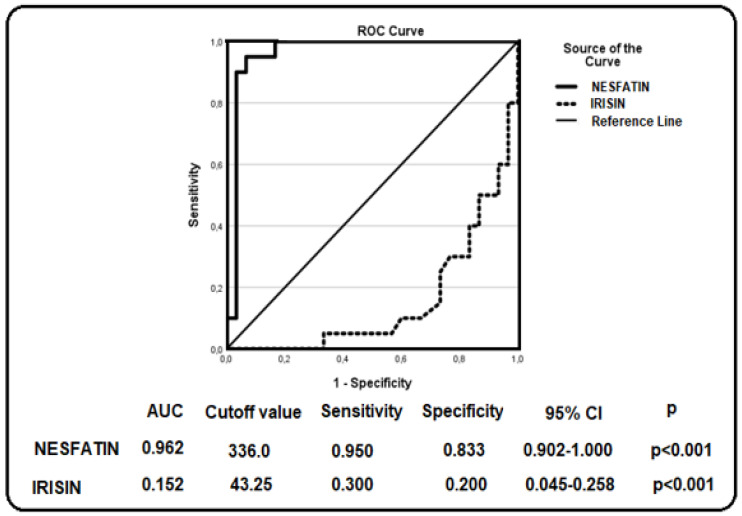
ROC curve analysis of nesfatin–1 and irisin in patients with HCI.

**Figure 2:**
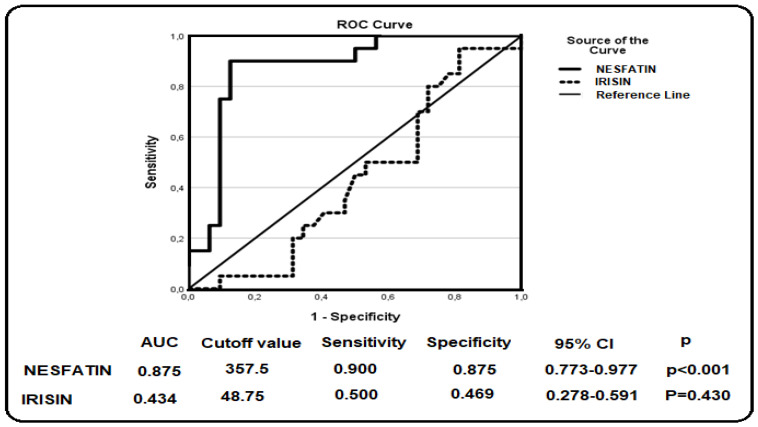
ROC curve analysis of nesfatin-1 and irisin in patients with CHC.

**Figure 3:**
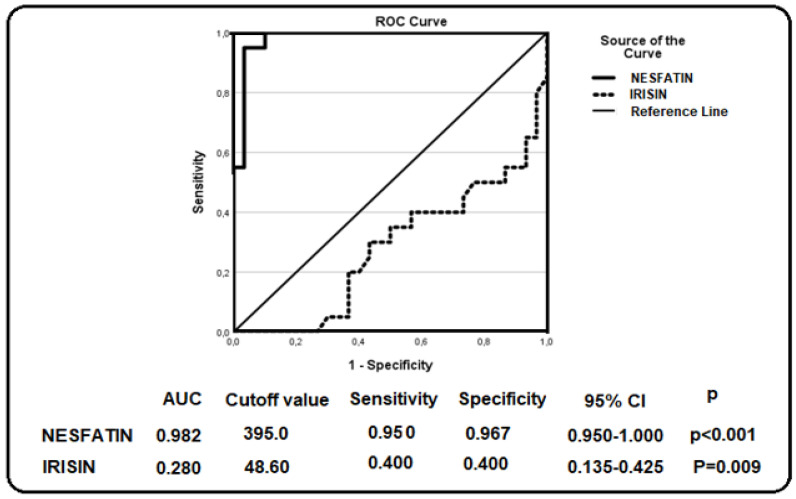
ROC curve analysis of nesfatin-1 and irisin in patients with CHB.

Receiver operating characteristic (ROC) curve analysis was performed to evaluate the diagnostic performance of the investigated biomarkers. The corresponding area under the curve (AUC) values, optimal cutoff points, sensitivity, specificity, 95% confidence intervals (CI), and *p*-values are presented in Figure 1, Figure 2 and Figure 3. These findings demonstrate the discriminatory capacity of the studied biomarkers across different patient groups.

Figure 1 presents the receiver operating characteristic (ROC) curve analysis evaluating the ability of nesfatin-1 and irisin to discriminate between patients with HBeAg-negative chronic infection (HCI) and healthy controls. Nesfatin-1 showed a relatively strong discriminative tendency, with an area under the curve (AUC) of 0.962, whereas irisin exhibited limited discriminative capacity (AUC = 0.152; *p* < 0.001 for both). Based on the ROC analysis, the optimal cutoff value was determined as 336.0 pg/mL for nesfatin-1 and 43.25 pg/mL for irisin.

Figure 2 presents the receiver operating characteristic (ROC) curve analysis evaluating the ability of nesfatin-1 and irisin to discriminate between patients with chronic hepatitis C (CHC) and healthy controls. Nesfatin-1 demonstrated a good level of discriminative performance, with an area under the curve (AUC) of 0.875 (*p* < 0.001), whereas irisin showed a relatively limited discriminative ability, with an AUC of 0.434 (*p* = 0.430). Nesfatin-1 demonstrated a relatively high discriminative ability, with an area under the curve (AUC) of 0.875 (*p* < 0.001), whereas irisin showed a limited discriminative ability, with an AUC of 0.434 (*p* = 0.430). Based on the ROC analysis, the optimal cutoff value was determined as 357.5 pg/mL for nesfatin-1 and 48.75 pg/mL for irisin.

Figure 3 presents the receiver operating characteristic (ROC) curve analysis evaluating the ability of nesfatin-1 and irisin to discriminate between patients with chronic hepatitis B (CHB) and healthy controls. Nesfatin-1 demonstrated a relatively high discriminative ability, with an area under the curve (AUC) of 0.982, whereas irisin showed a limited discriminative ability, with an AUC of 0.280 (*p* < 0.001 for nesfatin-1 and *p* = 0.009 for irisin). Based on the ROC analysis, the optimal cutoff value was determined as 395.0 pg/mL for nesfatin-1 and 48.60 pg/mL for irisin.

## 4. Discussion

Irisin was initially described as a myokine secreted by skeletal muscle in response to exercise and shivering, where its primary role was thought to be the promotion of white adipose tissue browning [20]. Irisin is also expressed in multiple tissues and has been suggested to exert a range of biological effects, including anti-inflammatory, anti-metastatic, neuroprotective, and antioxidative properties [20,21]. Irisin has been reported to exert anti-inflammatory effects by modulating inflammatory pathways and influencing the production of pro-inflammatory cytokines, possibly through the inhibition of the NF-κB pathway, which regulates interleukin-6 (IL-6) and tumor necrosis factor-alpha (TNF-α) production [22,23].

In hepatocellular carcinoma (HCC) cells, the irisin precursor FNDC5 has been implicated in the regulation of genes associated with lipogenesis, tumorigenesis, and inflammation. Increased irisin expression is thought to exert inhibitory effects on HCC development, possibly through the modulation of de novo lipogenesis [21].

Notch1 signaling, which has been implicated in hepatocellular carcinoma (HCC) progression and metastasis, has been well characterized in previous studies [24]. Positive associations have been reported between irisin and PRO-C3 and PRO-C6, biomarkers of collagen formation and fibrogenesis, suggesting a potential link with liver injury severity [25].

In two independent studies, Pazgan-Simon et al. reported lower circulating irisin levels in patients with cirrhosis and hepatocellular carcinoma (HCC) compared with healthy subjects [26,27]. Zhang et al. reported that preoperative irisin levels were significantly associated with postoperative complications following hepatectomy in patients with HCC [28].

Higher irisin levels in physically active individuals suggest that exercise contributes to metabolic regulation, potentially influencing pathways involved in obesity-associated liver disease and its complications [29].

Irisin levels have been shown to decrease in metabolic liver diseases. Lower circulating levels have been reported in individuals with obesity, NAFLD, and NASH compared to healthy controls [30,31]. 

Irisin is primarily a muscle-derived hormone closely associated with physical activity. Nutritional factors, including food intake and vitamin D status or supplementation, may influence circulating irisin levels, suggesting a potential interaction between nutrition and metabolic regulation [32,33,34].

In the present study, although irisin levels were lower across all patient groups compared to controls, statistical significance was observed only in the HCI group. This pattern suggests that the decrease in irisin may represent a general trend in chronic viral hepatitis, while the more pronounced reduction in the HCI group may reflect stage-specific alterations. Early metabolic or inflammatory changes in the HCI phase may contribute to this finding, whereas variability within other groups may have limited statistical significance. Therefore, irisin may still be involved in the pathophysiology of chronic viral hepatitis; however, its sensitivity to disease stage and intergroup variability should be taken into account.

Nesfatin-1 has predominantly been investigated in metabolic disorders, including obesity and metabolic liver diseases. Reduced circulating nesfatin-1 levels have been reported in individuals with non-alcoholic fatty liver disease (NAFLD), while alterations in nesfatin-1 levels have also been observed in obese populations [35,36]. In contrast, animal studies suggest that nesfatin-1 may exert protective effects, particularly through signaling pathways related to cellular survival and oxidative stress; however, the exact mechanisms remain incompletely understood [37]. Experimental studies have shown that nesfatin-1 may attenuate liver injury by reducing inflammation and oxidative stress [38]. Interestingly, a clinical study conducted in Turkey has reported elevated nesfatin-1 levels in individuals with liver cirrhosis compared with healthy controls [39].

Consistent with these findings, the present study demonstrated significantly higher serum nesfatin-1 levels in all patient groups (CHB, CHC, and HCI) compared with healthy subjects (*p* < 0.001). Moreover, ROC curve analysis suggested that nesfatin-1 may have a discriminative potential between infected patients and healthy individuals; however, its diagnostic utility should be interpreted cautiously due to the study design and sample size.

The consistent increase in nesfatin-1 levels across all patient groups may be explained by its role in inflammatory and stress-related pathways. Chronic viral hepatitis is characterized by persistent low-grade inflammation, which may stimulate nesfatin-1 secretion as part of a compensatory or protective response. Given its reported anti-inflammatory and antioxidative effects, elevated nesfatin-1 levels may reflect an endogenous attempt to counteract hepatic inflammation and oxidative stress. This pattern, observed across all disease groups, suggests that nesfatin-1 may serve as a more stable and generalized biomarker of chronic hepatic inflammation.

Recent studies have increasingly emphasized the role of metabolically active biomarkers, such as adipokines and myokines, in the pathogenesis of chronic liver diseases. Molecules including adiponectin, leptin, and fibroblast growth factor 21 (FGF21) have been shown to be involved in hepatic inflammation, fibrosis, and metabolic dysregulation [40]. These findings suggest that liver diseases are not solely local processes but are also closely linked to systemic metabolic alterations. The findings of the present study are also consistent with the literature supporting the role of metabolic peptides in the pathophysiology of liver diseases.

Taken together, these findings suggest that both irisin and nesfatin-1 may contribute to the pathophysiological processes of chronic viral hepatitis, even in the absence of cirrhosis or HCC. While previous studies have primarily focused on advanced liver disease, the present study provides evidence that these peptides may already be altered during the earlier stages of infection.

From a clinical perspective, the observed differential patterns of irisin and nesfatin-1 suggest that these biomarkers may reflect distinct aspects of disease pathophysiology. While nesfatin-1 appears to be a consistent indicator of chronic inflammatory burden, irisin may provide additional information regarding early metabolic alterations; however, the lack of direct comparison with established biomarkers and the absence of longitudinal data limit the immediate clinical applicability of these findings. Future studies integrating metabolic parameters and established clinical markers are needed to better define their diagnostic and prognostic roles.

This study has several limitations. The exclusion of patients with cirrhosis or hepatocellular carcinoma (HCC) limits the ability to evaluate changes in these biomarkers across different stages of disease progression. In addition, treatment response was not assessed; therefore, the potential roles of irisin and nesfatin-1 as markers for monitoring therapeutic response remain unclear. Another important limitation of this study is the lack of detailed assessment of lifestyle-related factors such as physical activity, dietary habits, and metabolic status. These variables may act as potential confounders affecting irisin and nesfatin-1 levels, and their effects cannot be completely excluded.

## 5. Conclusions

In conclusion, the findings of this study suggest that irisin and nesfatin-1 may be associated with the pathophysiology of chronic viral hepatitis and may reflect early disease-related changes. In particular, nesfatin-1 may demonstrate a notable ability to distinguish infected patients from healthy individuals.

However, these findings should be considered preliminary and exploratory in nature. Due to the single-center and cross-sectional design of the study, causal relationships between biomarker levels and disease status cannot be established. In addition, the absence of longitudinal data and direct comparison with established biomarkers limits the immediate clinical applicability of these results.

Therefore, further multicenter and longitudinal studies, including validation with independent cohorts and treatment follow-ups, are required to confirm these findings and to better define the diagnostic and prognostic roles of these biomarkers.

## Figures and Tables

**Table 1:** Demographic characteristics of the study groups.

Group	Sample Size (n)	Mean Age (Years)	Male:Female Ratio	BMI (kg/m^2^)
CHB	20	53.85	11:9	25.36
HCI	20	53.42	9:11	24.94
CHC	20	52.74	12:8	26.56
CG	30	52.23	15:15	24.72

Abbreviations: CHB, chronic hepatitis B; CHC, chronic hepatitis C; HCI, HBeAg-negative chronic hepatitis infection; CG, control group; BMI, body mass index.

**Table 2:** MANOVA results of irisin, nesfatin-1, and biochemical parameters among the study groups.

Parameter	df	F	*p*
Irisin (pg/mL)	3	9.634	<0.001
Nesfatin-1 (pg/mL)	3	47.339	<0.001
AST (U/L)	3	1.350	0.263
ALT (U/L)	3	1.343	0.266
GGT (U/L)	3	2.250	0.088
AFP (µg/L)	3	2.622	0.056
Total Bilirubin (mg/dL)	3	4.094	0.009
PLT (×10^9^/L)	3	3.337	0.023
PT/INR (%)	3	1.688	0.176

Abbreviations: df, degrees of freedom; F, F-statistic (MANOVA); *p*, *p*-value; AST, aspartate aminotransferase; ALT, alanine aminotransferase; GGT, gamma-glutamyl transferase; AFP, alpha-fetoprotein; PLT, platelet count; PT/INR, prothrombin time/international normalized ratio.

**Table 3:** Between-group comparisons based on MANOVA results.

Parameter	Group	Mean ± SD	F	*p*
Irisin (pg/mL)	CHB	42.95 ± 8.10	9.634	<0.001
	HCI	39.33 ± 7.62		
	CHC	47.46 ± 6.09		
	CG	49.79 ± 7.11		
Nesfatin-1 (pg/mL)	CHB	545.9 ± 80.61	47.34	<0.001
	HCI	482.4 ± 69.11		
	CHC	460.4 ± 97.33		
	CG	293.9 ± 71.75		
Total Bilirubin (mg/dL)	CHB	0.57 ± 0.23	4.094	0.009
	CHC	1.049 ± 0.68		
PLT (×10^9^/L)	CHC	211.4 ± 68.84	3.337	0.023
	CG	265.2 ± 53.18		
PT/INR (%)	–	–	1.688	0.176
AST (U/L)	–	–	1.350	0.263
ALT (U/L)	–	–	1.343	0.266
GGT (U/L)	–	–	2.250	0.088
AFP (µg/L)	–	–	2.622	0.056

Abbreviations: CHB, chronic hepatitis B; CHC, chronic hepatitis C; HCI, HBeAg-negative chronic infection; CG, control group; SD, standard deviation; F, F-statistic (MANOVA); *p*, *p*-value; PLT, platelet count; AST, aspartate aminotransferase; ALT, alanine aminotransferase; GGT, gamma-glutamyl transferase; AFP, alpha-fetoprotein; PT/INR, prothrombin time/international normalized ratio.

**Table 4:** Significant pairwise comparisons based on Scheffé post hoc test.

Parameter	Comparison	*p*
Irisin (pg/mL)	CHB–CG	0.017
	HCI–CHC	0.008
	HCI–CG	<0.001
Nesfatin-1 (pg/mL)	CHB–CHC	0.012
	CHB–CG	<0.001
	HCI–CG	<0.001
	CHC–CG	<0.001
Total Bilirubin (mg/dL)	CHB–CHC	0.017
PLT (×10^9^/L)	CHC–CG	0.050

Abbreviations: CHB, chronic hepatitis B; CHC, chronic hepatitis C; HCI, HBeAg-negative chronic infection; CG, control group; PLT, platelet count; *p*, *p*-value.

## Data Availability

The data supporting the findings of this study are not publicly available due to patient confidentiality but can be obtained from the corresponding author upon reasonable request.
